# Harnessing smartphone RGB imagery and LiDAR point cloud for enhanced leaf nitrogen and shoot biomass assessment - Chinese spinach as a case study

**DOI:** 10.3389/fpls.2025.1592329

**Published:** 2025-08-13

**Authors:** Aravind Harikumar, Itamar Shenhar, Miguel R. Pebes-Trujillo, Lin Qin, Menachem Moshelion, Jie He, Kee Woei Ng, Matan Gavish, Ittai Herrmann

**Affiliations:** ^1^ School of Materials Science and Engineering, Nanyang Technological University, Singapore, Singapore; ^2^ Singapore-HUJ Alliance for Research and Enterprise (SHARE), Singapore, Singapore; ^3^ The Robert H. Smith Faculty of Agriculture, Food and Environment, The Hebrew University of Jerusalem, Rehovot, Israel; ^4^ National Institute of Education, Nanyang Technological University, Singapore, Singapore; ^5^ Nanyang Environment and Water Research Institute (NEWRI), Singapore, Singapore; ^6^ School of Computer Science and Engineering, The Hebrew University, Jerusalem, Israel

**Keywords:** biomass estimation, smartphone, lidar, crop parameter modelling, smart farming

## Abstract

Accurate estimation of leaf nitrogen concentration and shoot dry-weight biomass in leafy vegetables is crucial for crop yield management, stress assessment, and nutrient optimization in precision agriculture. However, obtaining this information often requires access to reliable plant physiological and biophysical data, which typically involves sophisticated equipment, such as high-resolution *in-situ* sensors and cameras. In contrast, smartphone-based sensing provides a cost-effective, manual alternative for gathering accurate plant data. In this study, we propose an innovative approach to estimate leaf nitrogen concentration and shoot dry-weight biomass by integrating smartphone-based RGB imagery with Light Detection and Ranging (LiDAR) data, using *Amaranthus dubius* (Chinese spinach) as a case study. Specifically, we derive spectral features from the RGB images and structural features from the LiDAR data to predict these key plant parameters. Furthermore, we investigate how plant traits, modeled using smartphone data based indices, respond to varying nitrogen dosing, enabling the identification of the optimal nitrogen dosage to maximize yield in terms of shoot dry-weight biomass and vigor. The performance of crop parameter estimation was evaluated using three regression approaches: support vector regression, random forest regression, and lasso regression. The results demonstrate that combining smartphone RGB imagery with LiDAR data enables accurate estimation of leaf total reduced nitrogen concentration, leaf nitrate concentration, and shoot dry-weight biomass, achieving best-case relative root mean square errors as low as 0.06, 0.15, and 0.05, respectively. This study lays the groundwork for smartphone-based estimate leaf nitrogen concentration and shoot biomass, supporting accessible precision agriculture practices.

## 1 Introduction

Nitrogen is a cornerstone mineral nutrient essential for regulating the yield and overall health of crop plants, as it is a key component of plant amino acids, proteins, and nucleic acids. Subtropical leafy vegetable crops, such as spinach, are experiencing a growing global market demand due to their exceptional nutritional profile and relatively affordable price (Morelock and Correll, 2008). These crops respond notably well to nitrogen fertilization, attributed to their efficient nitrogen uptake mechanisms paired with less efficient nitrogen reductive systems (Cantliffe, 1972; Qiu et al., 2021). However, the nitrogen fertilizer use efficiency for leafy vegetable crops typically remains below 50% of the applied amount (Govindasamy et al., 2023; Valenzuela, 2024), reducing profitability and contributing to substantial nitrogen loss to the environment. Such loss leads to increased nitrogen runoff into water bodies and elevated greenhouse gas emissions (Zhao et al., 2012). Balancing crop nitrogen needs with fertilizer application is thus critical to optimize crop performance, enhancing nutritional quality, protecting the environment, and maximizing returns for farmers. Given nitrogen’s vital role in improving both nutritional value and market appeal of leafy vegetables, accurate estimation of leaf nitrogen concentration and shoot dry-weight biomass is essential for determining the specific nitrogen requirements of crop species and, consequently, for optimizing fertilizer application to maximize yield and profitability.

Traditional methods for assessing leaf nitrogen concentration, such as laboratory-based micro Kjeldahl analysis (Bradstreet, 1954), and for determining shoot dry-weight biomass through drying, rely on destructive sampling, making them impractical and costly in terms of time, financial resources, and labor. This necessitates the development of innovative approaches that allow timely, non-destructive estimation of these plant parameters. Smartphone sensing offers a non-invasive method for analyzing crop dynamics, presenting a promising pathway for phenological monitoring to improve crop management and optimize yields (Hufkens et al., 2019). Extensive research has demonstrated a robust relationship between Red-Green-Blue (RGB) imagery and leaf nutrient composition (Gausman, 1985; Lee and Lee, 2013). For example, studies indicate that leaf chlorophyll concentration, which correlates positively with leaf nitrogen levels, provides a reliable indicator of nitrogen concentration (Evans, 1983). The emergence of optical smartphone RGB imaging has thus provided a cost-effective approach for capturing crop traits compared to the use of expensive cameras. The widespread availability and affordability of smartphones have driven the development of methods that use smartphone-based RGB imagery to estimate leaf nitrogen concentration in staple crops, such as rice (Tao et al., 2020) and wheat (Chen et al., 2016). Although RGB imagery can provide estimates of leaf nitrogen by capturing variations in visible reflectance (Evans, 1983; Fan et al., 2022), its limited ability to capture three-dimensional structural information reduces its effectiveness for crop physiological trait estimation. While photogrammetry can offer some structural insights from RGB images, its accuracy depends on image quality, lighting, and scene complexity (Burdziakowski and Bobkowska, 2021). In contrast, Light Detection and Ranging (LiDAR) provides direct and highly accurate 3D measurements, making it better suited for estimating structural traits such as vegetation height, canopy span, and biomass Lefsky et al (2002). The recent integration of LiDAR sensors into smartphones marks a groundbreaking advancement, enabling the collection of structural plant data, including biomass estimation. Combining smartphone RGB data with LiDAR data offers potential for estimating both biophysical and physiological traits in leafy vegetable crops. For instance, Bar-sella et al. successfully estimated leaf transpiration in maize using smartphone RGB imagery and LiDAR data collected with an iPhone 13 Pro Max smartphone (Bar-Sella et al., 2024).

A comprehensive literature review reveals a significant research gap in the integration of relatively low-cost smartphone RGB and LiDAR data for estimating plant traits, including leaf nitrogen concentration and shoot biomass. Moreover, few studies have focused on nitrogen concentration and biomass estimation specifically for high-demand tropical leafy vegetables, such as Chinese spinach. Additionally, many of the existing such studies have been conducted in open-field conditions, which are subject to considerable variations by biotic and abiotic factors (Valenzuela, 2024; Chevalier et al., 2025; Hassona et al., 2024), whereas the current study focuses on semi-controlled tropical greenhouse conditions. Specifically, this study aims to achieve three main objectives: (a) to evaluate the effectiveness of combined smartphone-based RGB imagery and LiDAR data for accurately estimating leaf nitrogen concentration and shoot biomass in leafy vegetable crops within a semi-controlled greenhouse setting, (b) to quantify the relevance of the RGB and the LiDAR-based vegetation indices in **Tables 1**, **2** for estimating leaf nitrogen concentration and shoot dryweight biomass in Chinese spinach, and (c) to identify the optimal nitrogen dosage for maximizing crop dry-weight biomass yield using integrated smartphone RGB imagery and LiDAR data.

## 2 Materials

### 2.1 Study site, experimental setup and crop management

The experiment was conducted in a semi-controlled tropical greenhouse (Oasis Living Lab, Singapore), specifically designed for leafy vegetable production, from September to November 2023. The average temperature inside the greenhouse was 30.6°C, with a minimum of 24.5°C and a maximum of 45.1°C. The average humidity was 76.2%, ranging from a minimum of 43.9% to a maximum of 94.6%.

**Figure 1:**
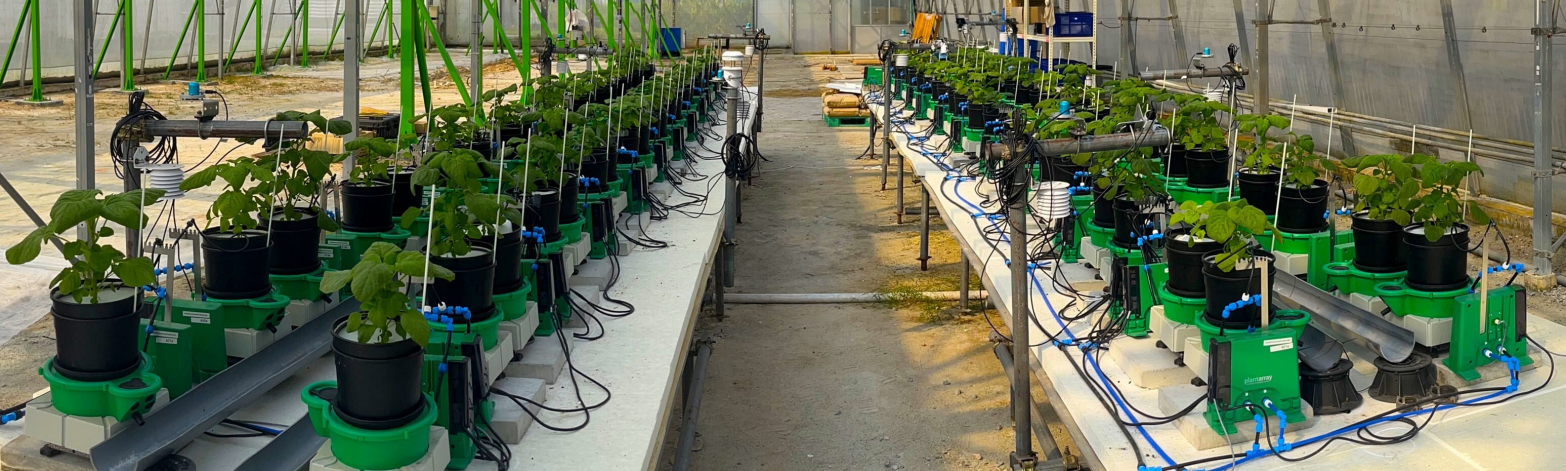
Experimental setup of 84 Chinese spinach plants in the semi-controlled greenhouse at the OasisLab, Singapore. Nitrogen dosing for each plant is precisely regulated using the PlantArray system, allowing controlled assessment of varying nitrogen treatments (in randomized block design) on plant growth and performance.

A total of 84 Chinese spinach plant species, known for their high productivity and nutritious value, were selected for this study (**Figure 1**). Seeds were initially sown in seedling trays and nurtured for 4 weeks to ensure optimal growth prior to transplantation (Shenhar et al., 2024). At the appropriate growth stage, the plants were transplanted into 4-L pots (Tefen Ltd., Nahsholim, Israel) filled with coco-peat substrate (Riococo Ltd., Sri Lanka) at the experiment’s commencement.

To evaluate the effects of different nitrogen dosages on plant growth and performance, plants were assigned to eight treatment groups, each consisting of 10 or 11 replicates. Each group received a specific nitrogen dosage - 20, 40, 80, 120, 160, 200, 400, or 800 parts per million (ppm) delivered in an aqueous solution. The recipe of the nutrient formulation is provided in **Table A1** section A.1 in **Appendix A**. Nitrogen fertigation was carefully regulated using precision pumps (Shurflo 5050-1311-H011, Pentair, MN, USA) controlled by the PlantArray system (Plant-Ditech, Yavne, Israel), as outlined in (Halperin et al., 2017). Irrigation was applied proportionally based on real-time transpiration measurements from the PlantArray system (Plant-Ditech, Yavne, Israel), with four brief irrigation events per night to maintain soil at field capacity and minimize variations in soil moisture content across treatments (Halperin et al., 2017).

**Figure 2:**
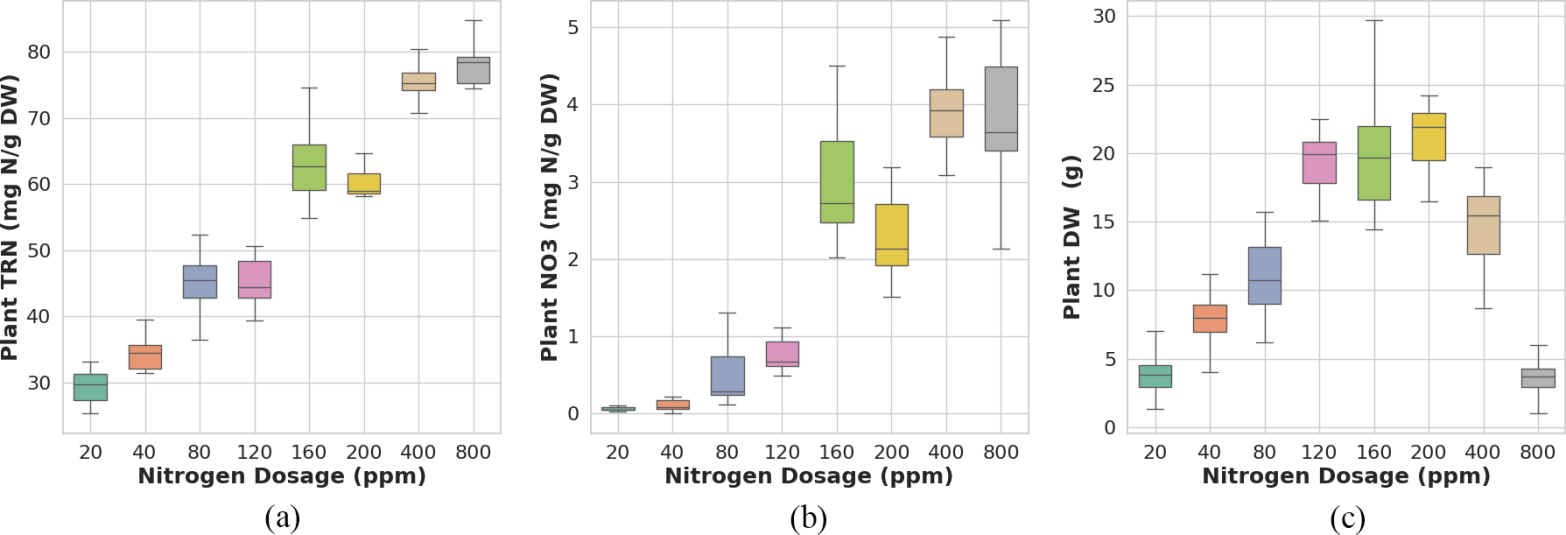
Reference data distribution for **(a)** plant total reduced nitrogen (*TRN*), **(b)** nitrate (*NO*_3_), and **(c)** dry weight (*DW*) in Chinese spinach across different nitrogen dosages.

The plants were cultivated for five weeks, from September 11 to October 17, 2023, prior to harvesting. Post-harvest, the shoots were manually separated into leaves and stems. The separated shoots were ovendried at 60°C for one week to ensure complete desiccation before laboratory analysis. The dried samples were then analyzed for leaf total reduced nitrogen (*TRN*) concentration, leaf nitrate (*NO*_3_) concentration, and shoot dry weight (*DW*) biomass. Here, *TRN* refers to the nitrogen content in its reduced forms, including nitrogen incorporated into amino acids, proteins, and other organic compounds, *NO*_3_ represents the inorganic nitrogen absorbed from the soil, and *DW* is an indicator of yield. Leaf nitrate ( $NO_{3}$ ) concentration was measured following (He et al., 2020). Dried leaf samples (0.01 g) were ground in 10 mL Milli-Q water, shaken at 37°C (200 rpm) for 2 hours, and filtered through a 0.45 m pore membrane using a vacuum filter. Quantification of $NO_{3}$ concentration was carried out using a Quikchem 8000 Flow Injection Analyzer (Lachat Instruments Inc., Milwaukee, WI, USA), with absorbance of the resulting magenta complex measured at 520 nm to derive the $NO_{3}$ concentration. TRN concentration was determined by digesting 0.05 g of dried sample with a Kjeldahl tablet in 5 mL of concentrated sulfuric acid at 350°C for 60 minutes, following the method of Allen et al. in (Allen, 1989). After digestion, TRN concentration was quantified using a Kjeltec 8400 analyzer (Foss Tecator AB, Höganäs, Sweden). The TRN concentrations, $NO_{3}$ concentrations, and the DW , of individual plants measured in the lab by destructive analysis of the harvest shoot tissue of individual plants are provided in **Table A2** in section A.2. The boxplots of the measured TRN , the $NO_{3}$ , and the DW parameter values for the different nitrogen dosage plant sets, are presented in **Figure 2**.

### 2.2 Smartphone data collection

**Figure 3:**
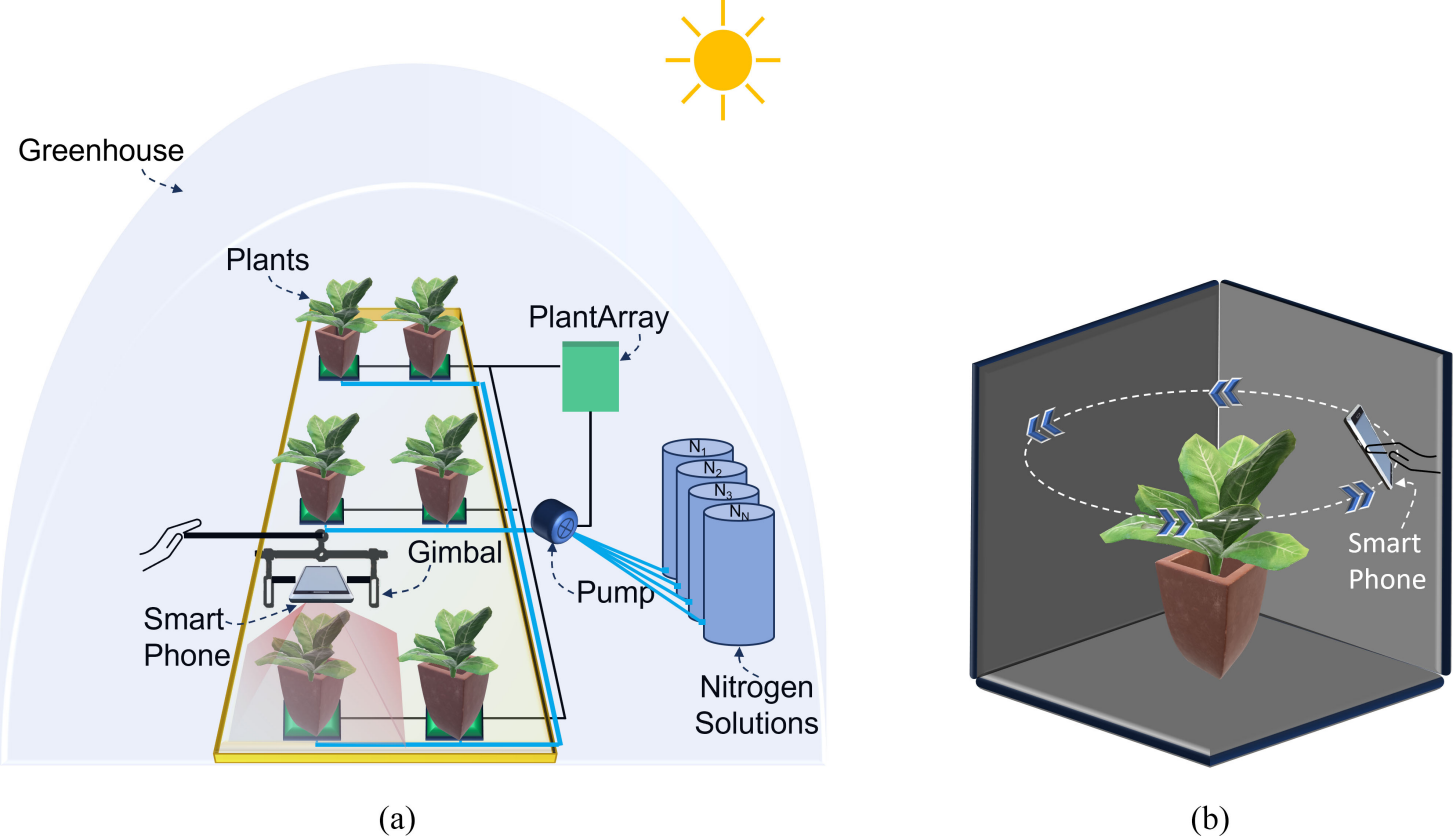
Schematic representation of the smartphone-based data acquisition setup in the indoor greenhouse. **(a)** RGB imagery acquisition setup showing the smartphone mounted on a gimbal platform and the PlantArray system managing nitrogen dosing for individual plants. **(b)** 3D LiDAR point cloud data acquisition setup, where the smartphone is maneuvered around the plant to capture detailed structural data of the plant crown.

**Figure 4:**
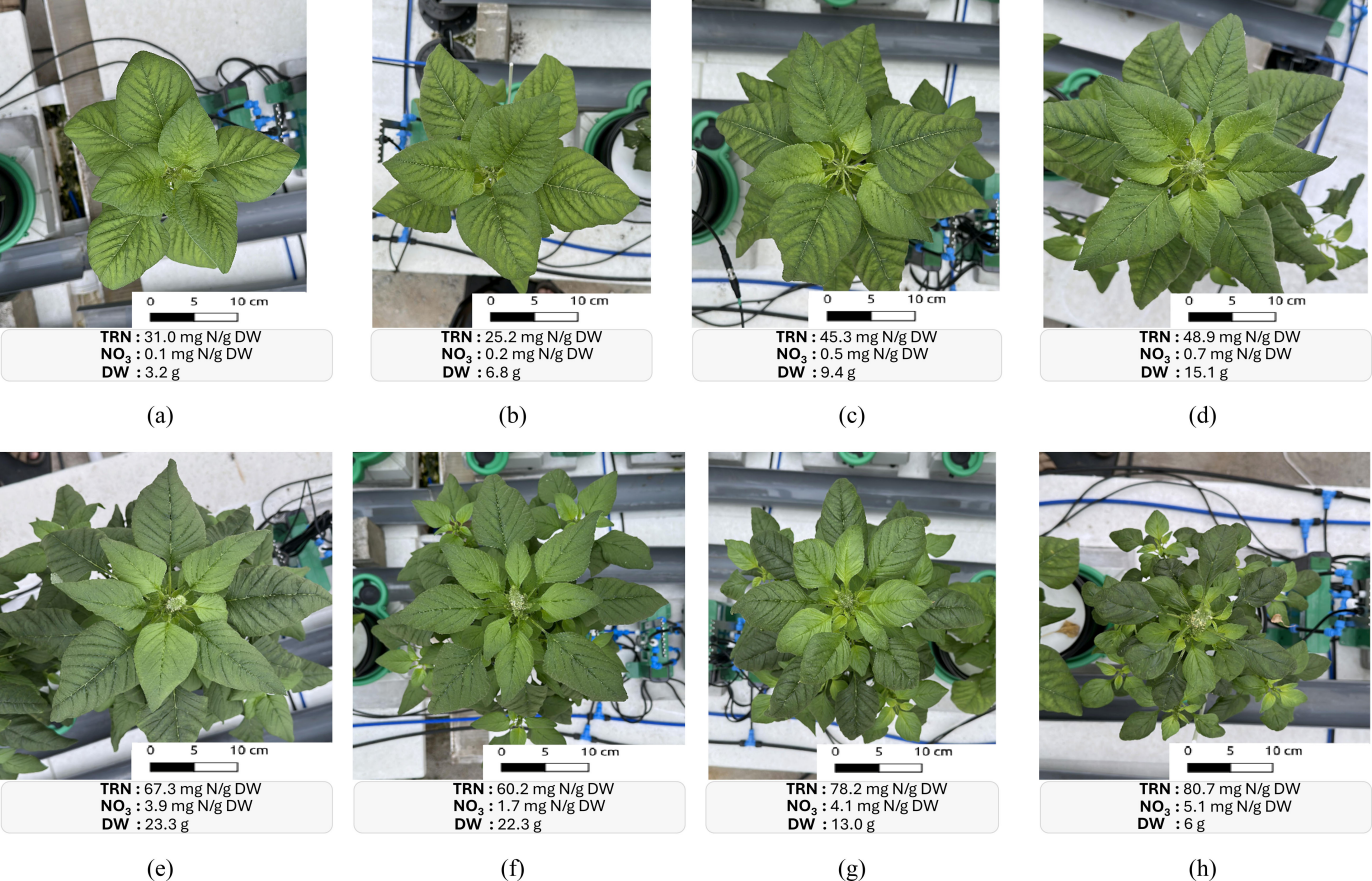
RGB images of Chinese spinach plants treated with varying nitrogen dosages: **(a)** 20 ppm, **(b)** 40 ppm, **(c)** 80 ppm, **(d)** 120 ppm, **(e)** 160 ppm, **(f)** 200 ppm, **(g)** 400 ppm, and **(h)** 800 ppm. The lab-based measurements of the leaf total reduced nitrogen (*TRN*) concentrations, the leaf nitrate (*NO*_3_) concentrations, and the shoot dry weight (*DW*) which is an indicator of yield for individual plants are reported at the bottom of the respective subfigure.

In this study, an iPhone 14 Pro Max smartphone (Apple, Cupertino, CA, USA) with a field of view (FoV) of 69°C, pixel size (PL) of 1.9 *µ*m, and focal length (F) of 26 mm was mounted on a manually held ZHIYUN Smooth 5 gimbal platform (Guilin Zhishen Information Technology Co., Ltd., Shenzhen, China) to acquire RGB imagery from a nadir perspective (**Figure 3a**). RGB images for all 84 Chinese spinach plants were captured within a 10-minute timeframe under clear skies, between 11:30 am and 12:00 pm, on the day preceding harvest (**Figure 4**). The RGB data acquisition height was set to ensure the entire plant crown fell within the camera’s field of view, specifically at 50 ± 5 cm above the highest point of the plant crown. Precise horizontal alignment and smartphone orientation during data capture were verified using the Pocket Bubble Level iPhone application (ExaMobile S.A., Bielsko-Biała, Poland) to maintain a level device position throughout the imaging process. At the end of data collection, an RGB image of a 99.9% circular white reference panel was taken to facilitate the conversion of raw RGB digital numbers to reflectance values.

Additionally, 3D LiDAR point cloud data of each of the 84 plants were captured in under 10 seconds using the iPhone 14 Pro Max’s in-built LiDAR sensor, operated via the Polycam 3D scanning application (Polycam, Inc., San Francisco, California). The LiDAR sensor specifications are consistent across iPhone 13 and iPhone 14 Pro models, allowing them to be used interchangeably for 3D data acquisition. Plants were positioned in direct sunlight against a dark background, 10–20 cm from the pot, to reduce background noise and enhance point cloud quality. The Polycam application’s LiDAR capture mode collected high-density point cloud data (i.e., 5–20 points per cm^3^). During LiDAR data acquisition, the smartphone was maneuvered in a circular path around and above each plant (**Figure 3b**) to obtain a comprehensive, detailed representation of the plant’s crown structure. The Polycam application generated the final raw 3D point cloud, with each point containing R, G, and B value attributes, within 20–30 seconds of scan completion and preprocessing initiation. Here, the decision to use LiDAR over photogrammetry was driven by its ability to directly capture accurate 3D structural information, even under challenging conditions, such as variable lighting and plant motion.

## 3 Methods

**Figure 5:**
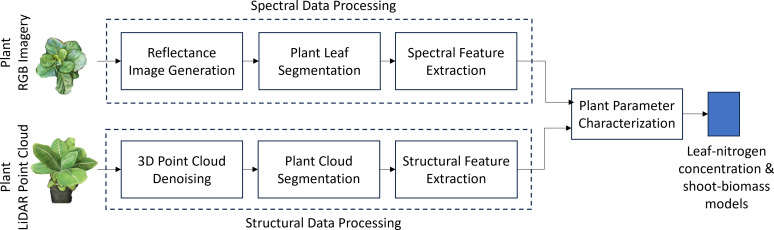
Block diagram of the smartphone-based leaf nitrogen concentration and shoot dry-weight biomass estimation setup for Chinese spinach. Spectral features derived from smartphone RGB imagery and structural features from LiDAR point cloud data are combined to estimate key leaf nitrogen concentration and shoot biomass.

The proposed approach characterizes physiological and biophysical crop traits by leveraging spectral and structural data obtained from smartphone RGB imagery and LiDAR point cloud data (**Figure 5**). In our case, the physiological trait of interest is DW , and the biophysical ones are TRN and $NO_{3}$ (as defined in 2.1). RGB imagery processing includes generating reflectance values to account for environmental variations in solar illumination, followed by leaf segmentation and extraction of spectral-reflectance features. Structural feature extraction from LiDAR data involves denoising and segmenting the 3D point cloud to isolate plant data from background objects and noise.

### 3.1 Spectral data processing

The inherent sensor-electronic noise present in the measured values was mitigated by performing dark image subtraction on the raw optical smartphone RGB images. A dark image was generated by covering the smartphone’s rear camera with a dark cloth. The resulting RGB images were then converted to reflectance values by dividing each digital number (DN) value by the average DN value from a 99.9% white reference image (Näsi et al., 2015).

**Figure 6:**
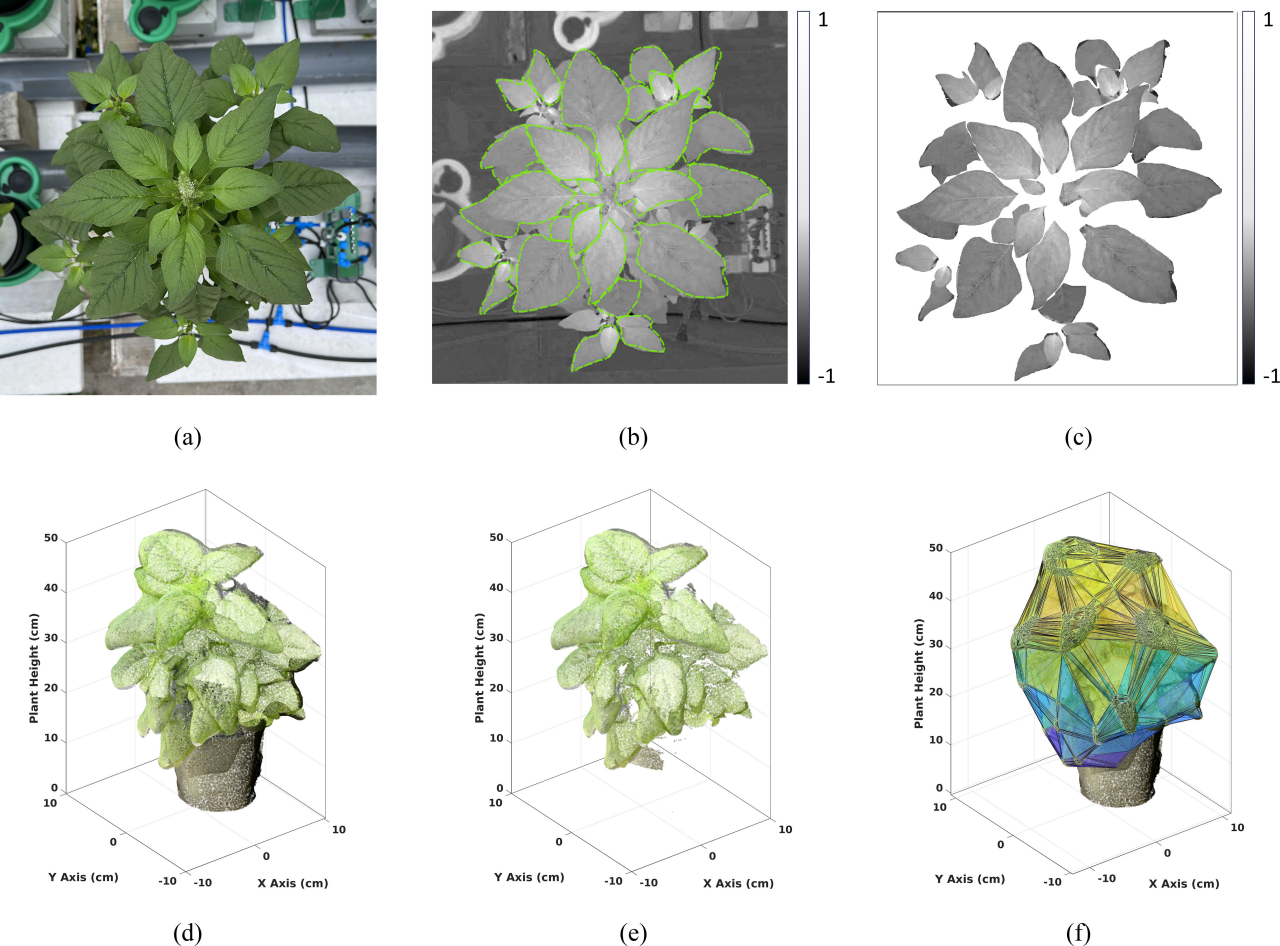
Visualization of Data Processing: (Top row): Top view **(a)** RGB image of a Chinese spinach plant captured using a smartphone. **(b)** Spectral index image, with boundaries delineated using Mask-CNN for leaf segmentation. **(c)** Resulting leaf-segmented image. (Bottom row): **(d)** 3D LiDAR point cloud of the plant, **(e)** filtered 3D point cloud after K-means clustering to isolate plant data, and **(f)** convex hull fitted to the filtered plant point cloud to extract structural features.

The individual RGB reflectance images captured during our experiment contained extraneous details, including background objects such as pots and other experimental hardware (**Figure 6a**). To minimize estimation errors, accurate delineation of plant leaves from the background was essential. We employed the Mask Convolutional Neural Networks (MaskCNN) algorithm (Zhang et al., 2021) for automatic leaf segmentation, chosen for its precision in identifying leaf structures (i.e., the objects of interest) within images. The CNN was trained on 245 individual leaf images, each carefully annotated by an expert from 150 randomly selected plant images. To enhance dataset size, diversity, and robustness, data augmentation techniques—such as rotations (45^°^, 90^°^ and 135^°^) and scalings (0.10, 0.25, 0.50, 0.75, and 1.25) - were applied to each original image, resulting in a total of 2,250 leaf samples for model training. The MaskCNN algorithm used distinct leaf traits such as shape, texture, and color to automatically extract leaf boundaries from the background (**Figure 6b**). The output of the MaskCNN segmentation process was a mask layer overlaying the original RGB reflectance image, where pixels corresponding to plant leaves were assigned a value of 1 and background pixels a value of 0. This binary mask effectively isolated the leaf regions from the background by multiplying it with the vegetation spectral index image, providing a clean extraction of the plant leaves in the vegetation spectral index imagery (**Figure 6c**).

To assess the accuracy of the MaskCNN-based leaf delineation, we utilized the Intersection over Union (IoU) metric (see Equation 1). This metric compares the estimated leaf mask against a manually delineated leaf mask. Let $A^{est}$ denotes the estimated leaf-mask area and $A^{ref}$ denotes the manually delineated leaf-mask area, then IoU is estimated as


(1) $$IoU=\frac{A^{est}\cap^{}A^{ref}}{A^{est}\cup^{}A^{ref}}$$

An IoU value of 1 indicates perfect overlap, while a value of 0 indicates no overlap (Thoma, 2016). MaskCNN segmentation performance was assessed using a set of 245 manually extracted leaves from 25 randomly selected plants across all nitrogen fertigation treatments.

The leaf regions identified by the MaskCNN segmentation algorithm were then extracted from the RGB reflectance images to estimate vegetation indices. These segmented leaf regions were used to calculate various vegetation indices that serve as proxies for different plant traits. The RGB spectral indices were quantitative measures derived from plant leaf reflectance values, reflecting their sensitivity to specific plant characteristics, such as chlorophyll content and overall vegetation vigor.

Chlorophyll concentration in leaves is a critical parameter for assessing plant greenness, as it reflects photosynthetic capacity, vigor, and shoot biomass (Pavlovica et al., 2014; Wagner, 2022). The Excess Green Index (ExG), Green Leaf Index (GLI), and Normalized Green-Red Difference Index (NGRDI) are commonly used to evaluate chlorophyll content in leaves (Rasmussen et al., 2016; Bassine et al., 2019). The ExG index, calculated by subtracting the red and blue reflectance from twice the green reflectance, is highly responsive to changes in chlorophyll concentration. The GLI measures vegetation “greenness” by assessing green reflectance relative to other wavelengths (Louhaichi et al., 2001). Similarly, the NGRDI, which normalizes the difference between green and red reflectance, also serves as an indicator of chlorophyll concentration.

In addition to chlorophyll content, certain indices are designed to evaluate other leaf characteristics, such as pigmentation, stress, and structure. For example, the Triangular Greenness Index (TGI) (Hunt et al., 2011) and Normalized Difference Yellowness Index (NDYI) (Sulik and Long, 2016) are valuable in this regard. Healthier leaves often have certain thicknesses and internal structures that influence light absorption and reflection; therefore, variations in TGI values can indicate differences in leaf morphology, such as thickness or density, affecting light interaction (Hunt et al., 2011). The NDYI, which measures the difference between green and blue reflectance, provides insights into yellow pigments, suggesting potential stress or senescence (Sulik and Long, 2016). Both indices are instrumental for monitoring vegetation health and detecting stress conditions (Barton, 2012; Zhang and Kovacs, 2012).

In this study, we used a total of 15 widely recognized RGB spectral indices from the literature. **Table 1** provides a detailed summary of these RGB spectral indices, including the parameters and calculations used to derive them from the smartphone-based RGB imagery. The average value of all leaf pixels were computed to obtain a plant-level RGB feature estimate for each index.

**Table 1:** The spectral indices derived from the smartphone RGB imagery of plants.

Index name	Index code	Formula	Reference
Green Leaf Index	GLI	(2G−R−B)/(2G+R+B)	Louhaichi et al. (2001)
Modified Green Red Vegetation Index	MGRVI	$\left(G^{2}-R^{2}\right)/\left(G^{2}+R^{2}\right)$	Bendig et al. (2015)
Red Green Blue Vegetation Index	RGBVI	(G−R∗B)/(G+R∗B)	Bendig et al. (2015)
Excess Green Index	ExG	2G−R−B	Woebbecke et al. (1995)
Excess Red Index	ExR	(1.4R−G)/(G+R+B)	Meyer and Neto (2008)
Triangular Greenness Index	TGI	$-0.5\left((\lambda_{R}-\lambda_{B}\right)\left(R-G\right)-\left(\lambda_{R}-\lambda_{G}\right)\left(R-B)\right)$	Hunt et al (2011)
Visible Atmospherically Resistant Index	VARI	(G−R)/(G+R-B)	Gitelson et al. (2002)
Normalized Red Index	NRI	R/(R+G+B)	Jia et al. (2004)
Normalized Green Index	NGI	G/(R+G+B)	Jia et al. (2004)
Normalized Blue Index	NBI	B/(R+G+B)	Jia et al. (2004)
Green Minus Red Index	GMRI	G−R	Jia et al. (2004)
Green Red Ratio Index	GRRI	G/R	Gamon and Surfus (1999)
Excess Green Minus Excess Red Index	EGMERI	ExGI−ExRI	Neto (2004)
Normalized Green Red Difference Index	NGRDI	(G−R)/(G+R)	Hunt et al. (2005)
Normalized Difference Yellowness Index	NDYI	(G−B)/(G+B)	Sulik and Long, 2016

R, G, B represent the reflectance values corresponding to the Red, Green, and Blue bands, with wavelengths λ*_R_*, λ*_G_*, and λ*_B_*, respectively.

### 3.2 Structural data processing

L et $P=\left\{\rho_{i}\in {\mathbb{R}}^{3}|i=1,2,\ldots ,M\right\}$ , with *ρ_i_* = (*x _i_, y_i_, z_i)_*, be the 3D LiDAR point cloud obtained using the smartphone (**Figure 6d**). Each point in the raw point cloud is composed of the three-dimensional space characterized by the x, y, and z Euclidean coordinates derived from the iPhone LiDAR sensor, together with the R, G, and B attribute values derived internally using the iPhone camera. However, the raw 3D point cloud often contains noisy points which arise from various sources such as suspended particles in the air, glitches in electronics, or errors in data acquisition and processing. To ensure accurate downstream analysis, it is essential to remove these noisy points from the point cloud. This denoising process involved several steps: a) voxelization: The volume covered by the 3D point cloud was divided into *S* regular cubic voxels {*V_i_* | *i* = 1, 2*,…, S*}, each with sides of equal length (i.e., 1cm), b) point density (PD) calculation: The number of points contained within each voxel was calculated, c) points with a voxel PD below a certain threshold were identified as noisy points and subsequently removed from the point cloud. The threshold value was chosen carefully to minimize both omission (failure to detect noisy points) and commission (incorrectly identifying valid points as noisy) errors. In our case, this threshold has been identified to be equal to 4 data points, i.e., we analyze all voxels i whose cardinality is greater than 4. By implementing this denoising procedure, the resulting point cloud was cleansed of isolated points, facilitating accurate structural parameter estimation (**Figure 6e**).

The subset of points $G=\left\{p_{j}|j=1,2,\ldots ,\;N\right\}\subset P$ which is part of the plant object was identified by using the two-class (i.e., *K* = 2) K-means clustering approach. This clustering method delineates the background data points from the plant data jointly using the R, G, and B attributes. By clustering the points into two distinct classes, one representing the black background and the other representing the green plant, we can effectively separate the vegetation from other objects in the scene (**Figure 6f**). This segmentation process played a crucial role in isolating the plant canopy from the surrounding environment, enabling more accurate analysis and interpretation of the structural parameters derived from the LiDAR point cloud.

Structural attributes encapsulating the physiological characteristics of the plant were derived from *G*, including plant height, maximum crown span, point density, and crown volume, providing valuable insights into the plant’s structural composition. The plant height (HT) was determined as the elevation of the highest point within the plant point cloud *G*. Meanwhile, the maximum crown span (CW) was calculated as the greatest distance between any two points within the point cloud *G* when projected onto the ground plane (X-Y plane), effectively capturing the lateral extent of the plant canopy. To assess the crown density (CD), we calculated the average point count within each 1cm voxel containing at least one point. This voxel-based approach provides a robust measure of crown density, offering insights into the spatial distribution of foliage within the plant canopy. By encapsulating the plant canopy within a convex envelope, an estimate of the plant’s crown volume (CV) is obtained, facilitating a comprehensive understanding of the vegetation structure Harikumar et al. (2017). The CV was estimated as the volume enclosed by the convex hull *V^Hull^* formed around *G* (**Figure 6f**). Here, the boundary fraction parameter *α* which controls the compactness of the convex hull, was set to 0.5 to produce a 3D hull that is neither too tight nor too loose. We acknowledge that convex hulls can overestimate canopy volume, particularly for sparse or irregular plant structures, as they enclose all outermost points. In this study, however, we focus on relative rather than absolute volume differences. Other structural traits included point count, point density and point count variance. The number of points above 75%, 50% and 25% of the plant height were represented as PC_75_, PC_50_ and PC_25_, respectively. The 9 plant-level LiDAR structural features used in the study are defined in **Table 2**.

**Table 2:** The structural indices derived from the smartphone LiDAR point cloud of plants.

Index name	Index code	Formula
Plant Height	HT	$\text{max}\left(\left\{p_{i}\left(z\right):p_{i}\in G\right\}\right)$
Crown Width	CW	$\text{max}(\left\{\text{Eucl}\left(p_{i}\left(x,y\right),p_{j}\left(x,y\right)\right):\begin{array}{ccc} p_{i} & \in & G\\ p_{j} & \in & G \end{array}\}\right)$
Crown Volume	CV	$V^{Hull}$
Point Count	PC	N
Point Density	PD	$N/V^{Hull}$
Point Count Variance	CR	$\mathrm{var}\left(V_{i}^{p}\right),i=1,\ldots ,S,V_{i}>0$
Point count above 75% height	PC_75_	$\left|\left\{p_{i}:p_{i}\left(z\right)>\text{percentile}\left(p_{i}\left(z\right),75\right)\right\}\right|,\;i=1,\ldots ,\;N$
Point count above 50% height	PC_50_	$\left|\left\{p_{i}:p_{i}\left(z\right)>\text{percentile}\left(p_{i}\left(z\right),50\right)\right\}\right|,\;i=1,\ldots ,\;N$
Point count above 25% height	PC_25_	$\left|\left\{p_{i}:p_{i}\left(z\right)>\text{percentile}\left(p_{i}\left(z\right),25\right)\right\}\right|,\;i=1,\ldots ,\;N$

*Eucl*(*A, B*) corresponds to Euclidean distance between points A and B. $V_{i}^{P}$ is the number of points in the *i^th^* voxel cell.

### 3.3 Plant traits characterization using regression modeling

In this study, plant traits were estimated using machine learning regression models based on spectral (**Table 1**) and structural (**Table 2**) features derived from RGB imagery and LiDAR point clouds. The spectral features primarily captured reflectance signals associated with leaf nitrogen content, while the structural features provided volumetric and height-related information essential for accurate biomass estimation. We employed three powerful regression techniques including the Support Vector Regression (SVR) (Vapnik, 2013), the Random Forest (RF) (Ho, 1995), and the Lasso Regression (Tibshirani, 1996), to model leaf nitrogen and dry weight biomass, using the structural and the spectral features. Each model was optimized via grid search and validated using cross-validation. A brief overview of each modeling approach is provided below, with their mathematical formulations included in section A.3 in **Appendix A**.

## 4 Results and discussion

Fifteen RGB spectral features were generated for individual Chinese spinach plants from RGB leaf data, which were first segmented using the MaskCNN algorithm as a preprocessing step. The MaskCNN algorithm achieved a high Intersection over Union (IoU) accuracy of 0.92 for the leaf segmentation. Additionally, nine LiDAR structural features were estimated from the plant point cloud segment identified through a 2-class K-means clustering algorithm.

**Figure 7:**
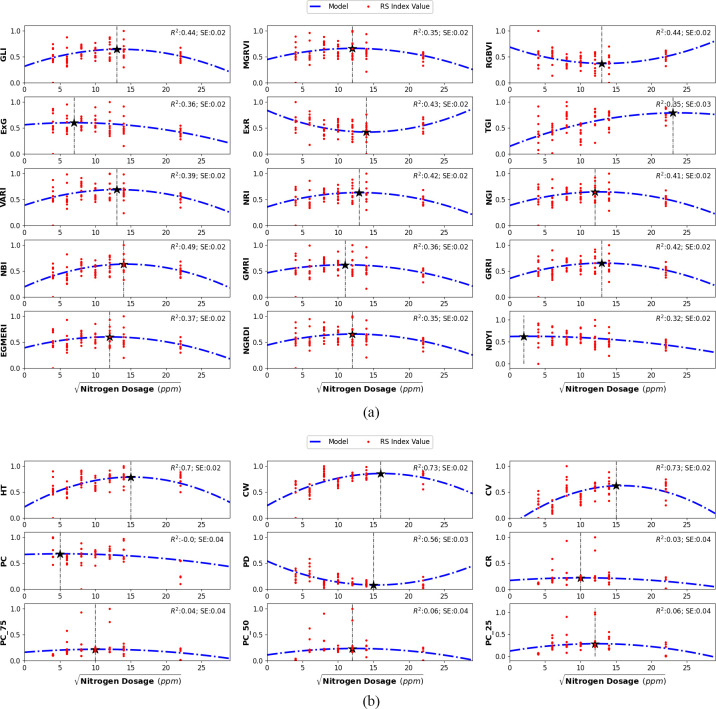
Second-order polynomial regression models illustrating the non-linear relationship between **(a)** RGB spectral features and **(b)** LiDAR structural features with nitrogen dosage in leaves. A square-root transformation was applied to the independent variable to normalize the initially skewed distribution. The minimum and maximum index values in each plot are indicated by the vertical dashed line. The Coefficient of Regression (*R* ^2^) and Standard Error (SE) are provided for each model.

Variations in nitrogen dosing affected all 24 features, each serving as a proxy for specific plant traits or overall stress indicators. For example, RGB spectral features such as the Visible Atmospherically Resistant Index (VARI) and the Modified Green-Red Vegetation Index (MGRVI) proxies for chlorophyll concentration and greenness, initially increased with nitrogen dosage but declined beyond a certain level. In contrast, indices like the Excess Red (ExR) and the Red-Green-Blue Vegetation Index (RGBVI), which are associated with plant stress, displayed an inverse relationship with nitrogen dosage. **Figure 7** illustrates the effect of nitrogen dosage on the RGB spectral and the LiDAR structural feature values. The visual assessments in **Figure 4** provide further insight into the effect of nitrogen treatments. Specifically, plants treated with both the lowest and highest nitrogen dosages exhibited reduced yields in terms of plant height and biomass, suggesting that extreme nitrogen levels may inhibit growth and development in Chinese spinach.

The relationship between nitrogen dosing and individual RGB spectral and LiDAR structural features was modeled using three types of regression models: linear, second-order polynomial, and third-order polynomial. In our case, the second-order polynomial regression model provided the best fit for all the features, effectively capturing the non-linear relationships between features and nitrogen dosage. Additionally, to address the initially skewed distribution of nitrogen dosages, a square-root transformation was applied, which normalized the nitrogen dosage distribution and improved the linearity of the modeled relationships. This transformation facilitated accurate estimation of plant parameters. **Figure 7** displays second-order polynomial models (dotted blue lines) that illustrate the relationships between various smartphone-derived indices and nitrogen dosages.

**Figure 8:**
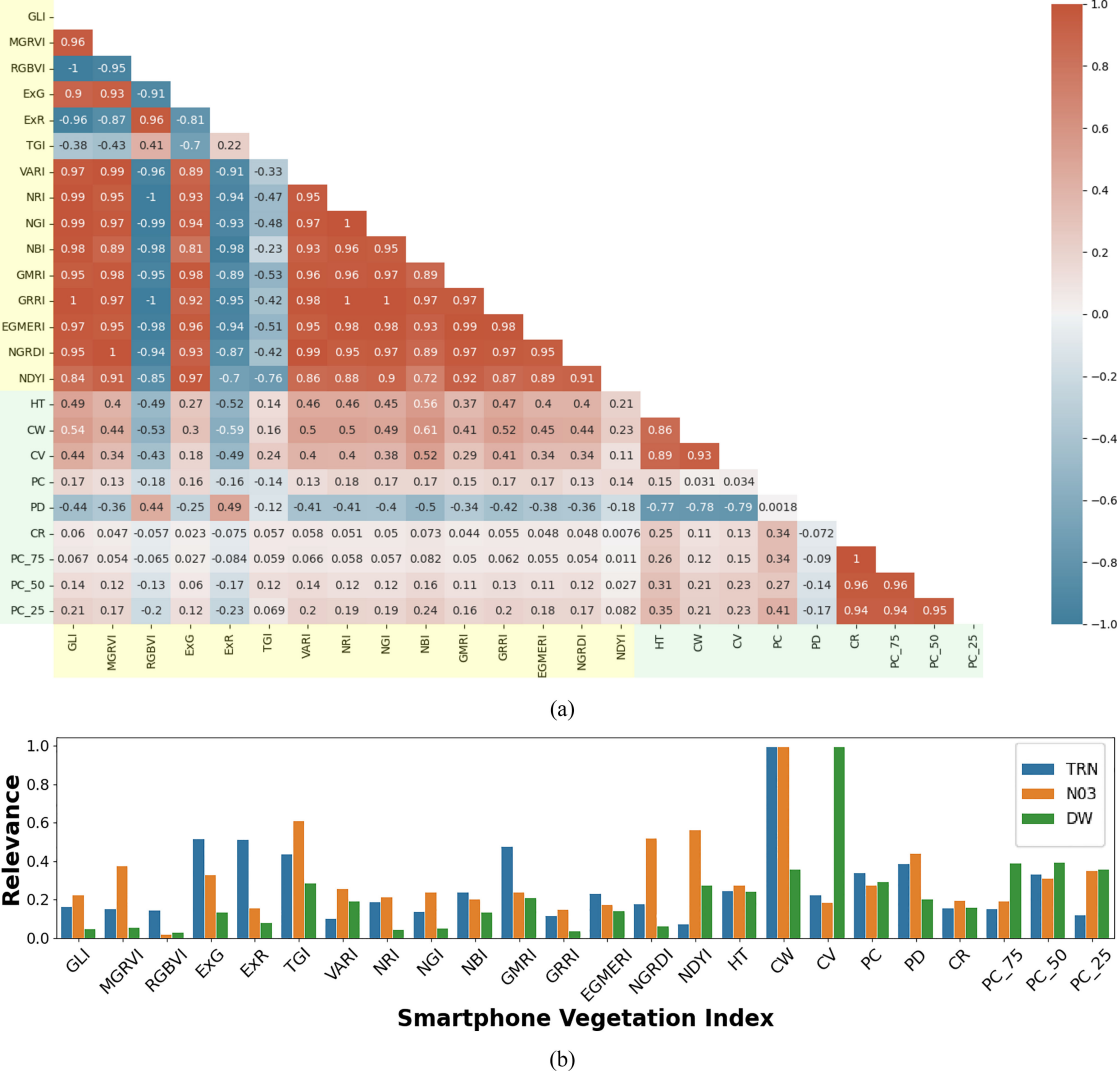
Multimodal feature correlation and relevance analysis for smartphone data: **(a)** Correlation matrix of RGB spectral indices (highlighted in light yellow) and LiDAR structural indices (highlighted in light green) derived from smartphone RGB imagery and LiDAR data. The matrix illustrates the relationships between different indices, with color intensity indicating the strength and direction of the correlation (red for positive, blue for negative correlations); **(b)** Normalized weights from the SVR model, indicating the relative importance of each RGB spectral and LiDAR structural feature in estimating leaf total reduced nitrogen (*TRN*), leaf nitrate (*NO*_3_), and shoot dry weight (*DW*).

In the case of spectral features, the high covariance observed was primarily due to the limited spectral bands captured by the RGB imagery (i.e., 3 bands). A similar pattern was evident in the structural features derived from LiDAR data, which also exhibit high inter-feature correlation. However, as shown in the correlation matrix (**Figure 8a**), spectral and structural features are generally uncorrelated, indicating their complementary nature. This allows them to capture distinct physiological signals relevant to modeling leaf nitrogen content and shoot dry-weight biomass. The low correlation between the two feature sets, alongside higher correlations within each set, confirms that they provide unique and complementary information. Hence, the joint use of RGB spectral and LiDAR structural features has the greater potential to produce accurate crop trait estimates.

We modeled the three plant parameters: leaf *TRN* concentration, leaf *NO*_3_ concentration, and shoot *DW* biomass, using three machine learning algorithms including the Support Vector Regression (SVR), the Random Forest (RF), and the Lasso Regression. Each model was trained on one of three feature sets: RGB spectral features only, LiDAR structural features only, or a combined set of both RGB and LiDAR features. This multi-configuration approach allowed us to evaluate the unique contributions of each feature set and their combinations in predicting key plant parameters. For all the regression frameworks, the total available labeled data were divided into training (70%) and testing (30%) sets. The accuracy assessment of the regression models was quantified using the Mean Absolute Error (MAE), Mean Error (ME), Root Mean Squared Error (RMSE), Relative Root Mean Squared Error (rRMSE), and Coefficient of Determination (R^2^) matrices (see equations A4-A8 in Section A.3 in **Appendix A**). Across all feature-set configurations, the RF model consistently performed well, yielding relatively lower Root Mean Squared Error (RMSE) and higher Coefficient of Determination (*R* ^2^) values compared to SVR and Lasso, particularly when using the combined feature set. Lasso Regression, however, showed high Mean Error (ME) and Mean Absolute Error (MAE) for the structural feature set (e.g., ME of 12.53 g), indicating possible overfitting or instability in Lasso’s performance for DW estimation. For the best-performing model (i.e., the combined set), the optimal hyperparameters were estimated as follows: for SVR, *C* = 10 and *∈* = 0.1; for RF, the number of decision trees *T* = 100 and maximum tree depth *D* = 5; and for Lasso regression, the regularization parameter λ = 0.01.

Further, a clear and consistent trend was observed across the feature sets: models trained only on the RGB spectral features resulted in the lowest prediction errors for *TRN* and *NO*_3_ estimation, while models trained only on the LiDAR-based structural features achieved the highest accuracy and lowest errors in predicting shoot *DW*. However, models trained using the combined set of both RGB and LiDAR features provided the lowest estimation errors (**Tables 3**, **4**). Overall, the results demonstrate the effectiveness of combining RGB imagery and LiDAR data in crop parameter modeling and highlight the robustness of the RF algorithm in managing complex multivariate data. The model estimation performance for *TRN* concentration, *NO*_3_ concentration, and shoot DW, quantified using MAE, ME, RMSE, rRMSE, and *R^2^* values, is provided in **Tables 3**, **4**.

**Table 3:** The *MAE*, *ME*, *RMSE*, *rRMSE*, and *R* ^2^ for leaf nitrogen concentration estimations done using the SVR, the RF, and the Lasso regression.

Parameter	Feature set	MLA	Accuracy estimates				
			*ME* ( $\frac{mgN}{gDW}$ )	*MAE* ( $\frac{mgN}{gDW}$ )	*RMSE* ( $\frac{mgN}{gDW}$ )	*rRMSE*	*R*²
*TRN*	**Spectral**	SVR	11.77	1.18	15.31	0.07	0.23
		RF	10.73	0.71	14.42	0.06	0.29
		Lasso	11.00	-0.01	14.10	0.06	0.33
	**Structural**	SVR	13.77	-1.71	17.01	0.08	0.15
		RF	10.91	1.24	15.19	0.07	0.22
		Lasso	13.75	-0.07	16.28	0.07	0.10
	**Combined**	SVR	10.64	-0.25	13.94	0.06	0.37
		RF	10.12	0.53	**13.38**	0.06	0.34
		Lasso	10.12	0.55	13.98	0.06	0.34
*NO*_3_	**Spectral**	SVR	1.16	-0.27	1.49	0.18	0.21
		RF	1.09	0.08	1.46	0.15	0.24
		Lasso	1.15	0.01	1.45	0.16	0.27
	**Structural**	SVR	1.46	-0.39	1.94	0.23	0.20
		RF	1.26	0.17	1.66	0.17	0.21
		Lasso	1.51	-0.03	1.84	0.21	0.15
	**Combined**	SVR	1.16	-0.15	1.52	0.17	0.26
		RF	1.09	0.07	**1.46**	0.15	0.27
		Lasso	1.13	0.01	1.47	0.16	0.29

The lowest RMSE values obtained among all the feature sets used for TRN and NO_3_ estimation are highlighted in bold

**Table 4:** The *MAE*, *ME*, *RMSE*, *rRMSE*, and *R* ^2^ for shoot *DW* estimation done using the SVR, the RF, and the Lasso regression.

Parameter	Feature set	MLA	Accuracy estimates				
			*MAE* (*g*)	*ME* (*g*)	*RMSE* (*g*)	*rRMSE*	*R*²
*DW*	**Spectral**	SVR	4.21	0.12	5.12	0.09	0.46
		RF	3.63	0.07	4.73	0.08	0.51
		Lasso	4.07	-0.04	5.06	0.09	0.45
	**Structural**	SVR	2.81	-0.65	3.58	0.06	0.73
		RF	2.27	-0.03	2.91	0.05	0.82
		Lasso	12.53	-0.12	3.18	0.05	0.78
	**Combined**	SVR	2.43	-0.44	3.08	0.05	0.80
		RF	2.15	0.04	**2.75**	0.05	0.84
		Lasso	2.29	-0.16	2.82	0.05	0.84

The lowest RMSE values obtained among all the feature sets used for TRN and NO_3_ estimation are highlighted in bold.

Beyond plant parameter estimation, we also focused on determining the optimal nitrogen dosage that maximizes plant chlorophyll and biomass using smartphone-derived RGB and LiDAR data. We consider optimal nitrogen dosage as the one that maximizes key traits such as leaf nitrogen and shoot biomass. These traits were assessed using RGB spectral indices and LiDAR-derived structural metrics, which provide reliable proxies for photosynthetic efficiency and overall plant growth. For example, as shown in **Figure 5a**, chlorophyll-sensitive spectral indices including ExR, VARI, and GMRI peak at approximately 149.3 ppm, indicating that this nitrogen level is generally associated with maximum chlorophyll concentration and photosynthetic efficiency Qiang et al. (2019). This supports the idea that the plant is able to utilize nitrogen most efficiently at this dosage. Additionally, LiDAR-derived metrics, including plant height, canopy volume, and estimates of dry weight, peak at 150.47 ppm, which corresponds to the nitrogen dosage that maximizes shoot dry-weight biomass, which is a key indicator of overall plant growth and vigor. By averaging these peak values, we derive an optimal nitrogen dosage of 149.9 ppm, which represents the most balanced nitrogen level for maximizing chlorophyll concentration, vigor, and shoot dry-weight biomass. This dosage of 149.9 ppm is close to the 120 ppm derived from the PlantArray system (Shenhar et al., 2024), providing further support for the validity of our smartphone-based approach. Although Shenhar et al. did not examine intermediate dosages between 120 ppm and 160 ppm, the alignment between these two studies strengthens the confidence in our findings, confirming that 149.9 ppm of nitrogen dosing provides the best overall plant vigor and shoot dry-weight biomass.

A detailed feature relevance analysis was conducted to assess the importance of various RGB and LiDAR-derived features in predicting key target variables: leaf *TRN* concentration, *NO*_3_ concentration, and *DW* biomass. Feature relevance was inferred based on the normalized weights assigned to each feature during SVR model training. These normalized weights were averaged across 100 modeling iterations, with each iteration using randomly generated training and test datasets to ensure robustness. The results indicate that feature importance varied significantly across models, reflecting the specific demands of each prediction task. For example, models developed to predict *TRN* and *NO*_3_ concentration assigned higher importance to the RGB features, likely because these spectral indices are closely related to nitrogen content, which affects reflectance in the visible spectrum. In contrast, models estimating shoot *DW* generally assigned greater weight to structural features obtained from LiDAR data, reflecting that biomass estimation can be more reliably modeled using plant morphology rather than spectral characteristics. Specifically, *DW* estimation showed high reliance on LiDAR-derived structural indices such as crown volume (*CV)* and point count at 75% height (*PC*_75_), which describe the plant’s physical structure and distribution. Conversely, for *TRN* and *NO*_3_ estimation, the models placed more emphasis on spectral features such as the Triangular Greenness Index (*TGI*) and Excess Green (*ExG*), both closely related to chlorophyll content and leaf greenness. These findings suggest a clear pattern: LiDAR-derived structural features are more effective for estimating morphological traits like biomass, while RGB features are better suited for predicting physiological traits like nitrogen and chlorophyll concentration. This distinction underscores the complementary nature of smartphone RGB imagery and LiDAR data in crop parameter modeling, with each sensor type contributing uniquely to different aspects of plant structure and function (**Figure 8b**).

To evaluate our methodology, we compared its performance with a leading state-of-the-art leaf nitrogen concentration estimation approach (SoA-NC) that relies solely on smartphone RGB imagery. This benchmark method uses the Dark Green Color Index (*DGCI*), derived from intensity, hue, and saturation metrics of the plant leaves (Paleari et al., 2022). However, using only a single RGB image feature index limits its accuracy in estimating leaf nitrogen concentration. Our results illustrate the superior performance of our models, which consistently produced more accurate and precise estimates of leaf *TRN* and *NO*_3_ concentrations compared to the SoA-NC method. This improvement highlights the advantage of integrating advanced machine learning algorithms with both spectral and structural features derived from smartphone RGB and LiDAR sensors. Unlike the SoA-NC method, which focuses solely on spectral data, our approach leverages the complementary strengths of RGB features (e.g., color indices) and LiDAR structural features (e.g., plant height and canopy shape). Whatsoever, high variance in individual feature index values, even within a nitrogen treatment group, due to non-uniform lighting or shading on plant leaves, is a known source of error, which manifests as significant variance in *TRN*, *NO*_3_ and *DW* estimates. Additionally, certain LiDAR-derived features based on point count and point density exhibited low $R^{2}$ values, likely due to the limited variation in these features across the sampled plants.

We also compared shoot *DW* estimation performance with a state-of-the-art leaf-area-based shoot *DW* estimation (SoA-DW) method (Li et al., 2020). The SoA-DW method estimates shoot *DW* by: (a) segmenting plant leaves using Otsu’s thresholding on the green channel, (b) calculating leaf area (*LA_p_*) as the product of the total number of plant pixels (*PPN*) in the image, the individual pixel length (*PL*) of the phone camera, and the magnification factor (*MF*) of the phone camera, and (c) estimating shoot *DW* using a linear model based on *LA_p_* and actual *DW* values obtained from destructive sampling. Our proposed shoot *DW* estimation method more accurately tracked actual shoot *DW* compared to the SoA-DW method. The reduced performance of the SoA-DW method likely results from its inability to capture plant height from images and from errors in leaf delineation due to non-uniform lighting and shading.

**Figure 9:**
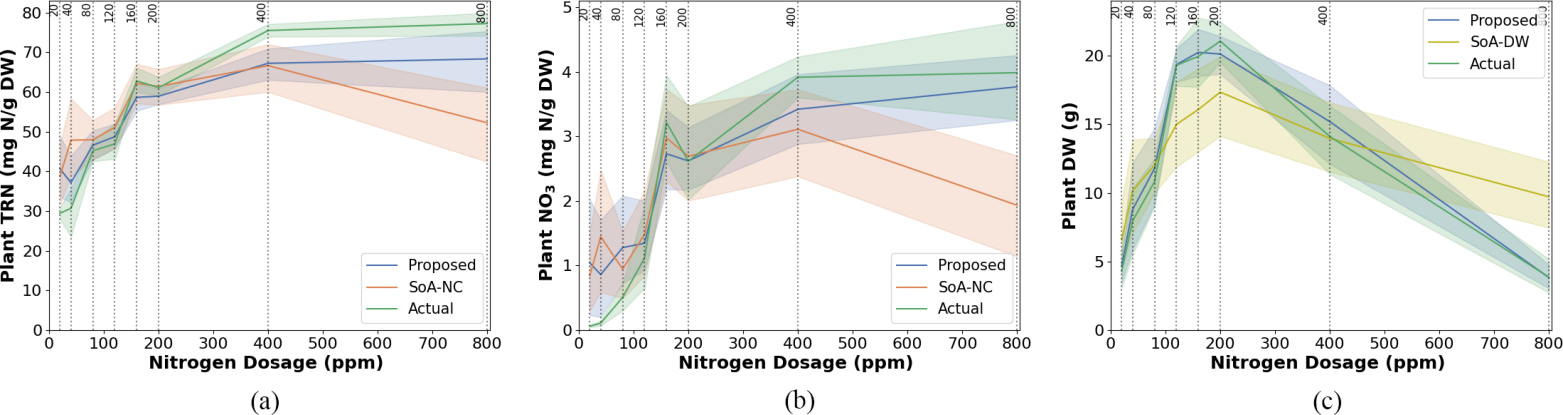
Model estimation performance for **(a)** total reduced nitrogen (*TRN*), **(b)** nitrate (*NO*_3_), and **(c)** dry weight (*DW*) in Chinese spinach under varying nitrogen dosages. Each subfigure compares the performance of the proposed estimation method against state-of-the-art (SOA) estimation approaches, with reference data obtained from destructive leaf analysis. The SoA methods for nitrogen concentration and dry weight are denoted as SoA-NC and SoA-DW, respectively. Standard errors (SE) are shown as shaded regions around the model lines.

A direct comparison of the *TRN*, *NO*_3_, and *DW* estimates obtained from our non-destructive model with those derived from destructive sampling method is presented in **Figure 9**, providing validation of the model’s accuracy and reliability. The results demonstrate that the proposed handheld smartphone-based system can accurately estimate leaf *TRN*, leaf *NO*_3_, and shoot *DW* without the need for manual harvesting, outperforming state-of-the-art methods that rely solely on RGB data.

## 5 Summary and conclusion

Smartphone-based RGB imagery and LiDAR data show strong potential for modeling the leaf nitrogen and shoot dry-weight biomass of *Amaranthus dubius* (Chinese spinach), serving as a case study for subtropical leafy vegetable crops. The combined use of the spectral and the structural features derived from smartphone-acquired RGB and LiDAR data, respectively, enables accurate estimation of leaf *TRN*, *NO*_3_, and *DW* in Chinese spinach, with RMSE values as low as 3.9 mg N/g DW, 1.46 mg N/g DW, and 2.7 g, respectively. Spectral indices were found to be more effective for estimating leaf nitrogen concentration, whereas structural indices provided better performance for shoot biomass estimation. The optimal nitrogen dosage for maximizing yield and vigor in Chinese spinach was determined to be 149.9 ppm. The encouraging results from this study demonstrate the strong potential of using smartphones for yield management in leafy vegetable crops. Planned future works include a) investigating the effectiveness of the proposed methodology on other leafy vegetable crops such as the Brassica oleracea (Chinese broccoli), b) using different generalized linear and or additive machine learning modeling techniques, and c) expanding the application of this methodology to assess plant requirements for other essential mineral nutrients.

## Data Availability

The raw data supporting the conclusions of this article will be made available by the authors, without undue reservation.

## A APPENDIX A

### A.1 Nutrient Recipe

The composition of the different nutrient solutions used for nitrogen fertigation treatments for the 84 Chinese spinach plants is shown in **Table A1**.

**Table A1:** Composition of the different nutrient solutions used for the nitrogen fertigation treatments.

Ingredients	Nitrogen fertilizer dosages (ppm or mg/L)							
	20	40	80	120	160	200	400	800
KNO_3_ (mg/L)	34.000	34.000	34.000	34.000	34.000	34.000	34.000	34.000
K_2_SO_4_ (mg/L)	150.000	150.000	150.000	150.000	150.000	150.000	150.000	150.000
NaNO_3_ (mg/L)	0.000	0.000	0.000	250.000	360.000	360.000	360.000	360.000
NH_4_NO_3_ (mg/L)	40.000	98.000	212.000	212.000	277.000	391.000	964.000	2110.000
MgSO_4_·0H_2_O (mg/L)	150.000	150.000	150.000	150.000	150.000	150.000	150.000	150.000
C_10_H_12_N_2_NaFeO_8_ (mg/L)	18.000	18.000	18.000	18.000	18.000	18.000	18.000	18.000
MnSO_4_·1H_2_O (mg/L)	5.500	5.500	5.500	5.500	5.500	5.500	5.500	5.500
ZnSO_4_·7H_2_O (mg/L)	1.320	1.320	1.320	1.320	1.320	1.320	1.320	1.320
CuSO_4_·5H_2_O (mg/L)	0.496	0.496	0.496	0.496	0.496	0.496	0.496	0.496
H_3_BO_4_ (mg/L)	0.217	0.217	0.217	0.217	0.217	0.217	0.217	0.217
MoO_3_ (mg/L)	0.131	0.131	0.131	0.131	0.131	0.131	0.131	0.131
CaSO_4_·2H_2_O (mg/L)	217.000	217.000	217.000	217.000	217.000	217.000	217.000	217.000
H_3_PO_4_ (µL/L)	60.000	60.000	60.000	60.000	60.000	60.000	60.000	60.000

### A.2 Destructive Sampling Results: Leaf Nitrogen and Shoot Dry-weight Biomass Traits

The TRN concentrations in the leaves, the NO_3_ concentrations, and the DW were measured in the laboratory through destructive sampling of the post-harvest shoot tissue for individual plants, are reported in **Table A2**.

**Table A2:** The leaf total reduced nitrogen (*TRN*) concentrations, the leaf nitrate (*NO*_3_) concentrations, and the shoot dry weight (*DW*) measured in lab by destructive analysis of the post-harvest shoot tissue, for the individual plants.

Plant_ID	Nitrogen dosage (ppm)	*TRN* ( $\frac{mgN}{gDW}$ )	*NO*_3_ ( $\frac{mgN}{gDW}$ )	*DW*(*g*)
A01a	40	25.23	0.219	6.800
A01b	120	48.910	0.718	15.100
A02a	800	78.200	3.400	1.000
A02b	80	52.400	0.289	6.200
A03a	200	64.630	3.190	21.900
A03b	400	76.860	3.846	15.000
A04a	160	65.570	2.850	21.100
A04b	20	29.860	0.066	4.100
A05a	120	44.450	0.645	20.100
A05b	800	65.710	2.131	2.900
A06a	20	32.040	0.092	1.300
A06b	200	58.900	2.085	19.400
A07a	40	39.460	0.172	4.000
A07b	160	58.970	2.618	15.900
A08a	80	45.280	0.575	9.400
A08b	400	80.410	4.000	5.700
A09a	400	78.160	4.100	13.000
A09b	800	74.450	3.258	1.500
A10a	40	34.870	0.193	8.100
A10b	120	39.370	1.082	15.200
A11a	80	43.930	0.905	8.600
A11b	160	56.560	2.725	18.700
A12a	200	60.250	1.727	22.300
A12b	20	31.030	0.096	3.200
A13a	20	29.150	0.047	2.600
A13b	160	60.280	2.336	17.300
A14a	200	61.190	2.008	23.400
A14b	80	51.400	1.305	11.600
A15a	40	31.500	0.158	9.100
A15b	400	75.550	4.232	12.500
A16a	120	50.640	0.600	19.800
A16b	800	78.660	3.896	4.200
A17a	20	29.480	0.049	4.400
A17b	120	69.130	4.940	22.500
A18a	80	46.510	1.014	12.700
A18b	400	74.970	3.275	8.700
A19a	160	59.180	2.260	29.700
A19b	800	84.790	6.692	3.800
A20a	40	33.890	0.094	11.200
A20b	200	58.280	1.817	21.200
A21a	120	45.330	1.108	20.400
A21b	200	59.040	2.850	16.500

### A.3 Regression Models

The SVR model with the linear kernel (Vapnik, 2013) is used to accurately model the plant parameters, for its exception ability to model underlying relationship and quantifying feature relevance. Equation A1 shows the objective function and constraints of SVR with the linear kernel.

L et *x _k_* ∈ *R^F^* be a *F* dimensional feature vector of the *k^th^* training sample, *y_k_* be the target value corresponding to the *k^th^* training sample, *w* = [*w*_1_ *, w*_2_ *,…, w_F]_* be the vector containing the scalar weights associated with individual features in the feature set, *b*be the bias term, *∈* be the margin of tolerance,

$\xi_{k}^{*}$ and ξk be the slack variables associated with each data point, and C be the regularization factor. The SVR is mathematically formulated as

A1
$$\begin{array}{l} \text{Minimize }J\left(w,\xi ,\xi^{*}\right)=\frac{1}{2}w^{T}w+C\sum_{k=1}^{N}(\xi_{k}+\xi_{k}^{*}),\\ \text{Subject}\;\text{to}:\\ y_{k}-\left(w^{T}x_{k}+b\right)\leq є+\xi_{k}{\text{∀}}_{k},\\ \left(w^{T}x_{k}+b\right)-y_{k}\leq є+\xi_{k}^{*}{\text{∀}}_{k},\\ \xi_{k}^{*},\xi_{k}\geq 0{\text{∀}}_{k} \end{array}$$

Here, the parameters *C* and *є* were estimated using grid search. The value of *C* was varied over the range [0.01, 0.1, 1, 10, 100], while *є* was varied over the range [0.01, 0.1, 0.5, 1], employing 5-fold crossvalidation to assess model performance. The model fitting was repeated 100 times using different random sets of training and testing data, and the individual parameter estimates $\hat{y_{k}}=\left(w^{T}\phi \left(x_{k}\right)+b\right)$ were averaged to obtain a reliable target parameter estimate.

The RF regression (Ho, 1995) follows an ensemble learning approach, where a collection of decision tree regression models operates independently to generate parameter estimates. The predictions from these individual trees are then averaged to produce a more reliable estimate. If, the RF model consists of *T* individual decision tree regression functions *f _t_* (*x*) where *t* = 1, 2*,…, T*, the target parameter estimate $\hat{y_{k}}$ is calculated as the average of estimates obtained from the individual trees using Equation A2.

A2
$$\begin{array}{l} \hat{y_{k}}=\frac{1}{T}\sum_{t=1}^{T}f_{t}\left(x_{k}\right) \end{array}$$

Here, the *T* and tree depth *D* of each tree were estimated using grid search on *T* for the range [1, 200] with a step size of 10, and *D* for the range [3, 5, 7, 10, 15] utilizing 5-fold cross-validation with, to assess the model performance.

We also model the plant parameters using the Lasso Regression (Tibshirani, 1996) which is a widely used linear regression technique that incorporates feature regularization. Let, *y_k_* is the true target variable value, *X_k_* be the vector of predictors for the *k^th^* observation, *w* be the vector of coefficients to be estimated, and λ *>* 0 be the regularization parameter that controls the strength of the penalty. Then, Lasso Regression is mathematically represented as

A3
$$\begin{array}{l} \text{Minimize }L\left(w,\beta \right)=\sum_{i=1}^{N}{\left(y_{k}-w^{T}x_{k}\right)}^{2}+\text{λ}\sum_{j=1}^{F}|\left|\beta_{j}\right|{|}_{1} \end{array}$$

The *λ* was set by performing grid search on the range [0.01,0.1,1,10,100]. Once (A3) is optimized, the target variable estimate for test data points *t* is obtained as ${\hat{y}}_{t}=w^{T}x_{t}$ .

A4
$$\begin{array}{l} \text{ME}=\frac{1}{N}\sum_{k=1}^{N}\left(y_{k}-{\hat{y}}_{k}\right) \end{array}$$

A5
$$\begin{array}{l} \text{MAE}=\frac{1}{N}\sum_{k=1}^{N}\left|y_{k}-{\hat{y}}_{k}\right| \end{array}$$

A6
$$\begin{array}{l} \text{RMSE}=\sqrt{\frac{1}{N}\sum_{k=1}^{N}{\left(y_{k}-{\hat{y}}_{k}\right)}^{2}} \end{array}$$

A7
$$\text{rRMSE}=\frac{RMSE}{\bar{y}}$$

A8
$${\text{R}}^{2}=1-\frac{\sum_{k=1}^{N}{\left(y_{k}-{\hat{y}}_{k}\right)}^{2}}{\sum_{k=1}^{N}{\left(y_{k}-\bar{y}\right)}^{2}}$$

where, *y* is the true response, $\hat{y}$ is the estimated response, and $\bar{y}$ is the sample mean.
